# Supplementary material for: Nanoscopic Approach to Study the Early Stages of Epithelial to Mesenchymal Transition (EMT) of Human Retinal Pigment Epithelial (RPE) Cells In Vitro
Source: Life (Basel). 2020 Jul 30;10(8):128. doi: 10.3390/life10080128 (PMC7460373; doi:10.3390/life10080128)
Supplement: Supplementary file 1 [file life-10-00128-s001.pdf]

life

Supplementary Materials of “Nanoscopic Approach to Study Early Stages of Epithelial to Mesenchymal Transition (EMT) of Human Retinal Pigment Epithelial (RPE) Cells *in vitro*”

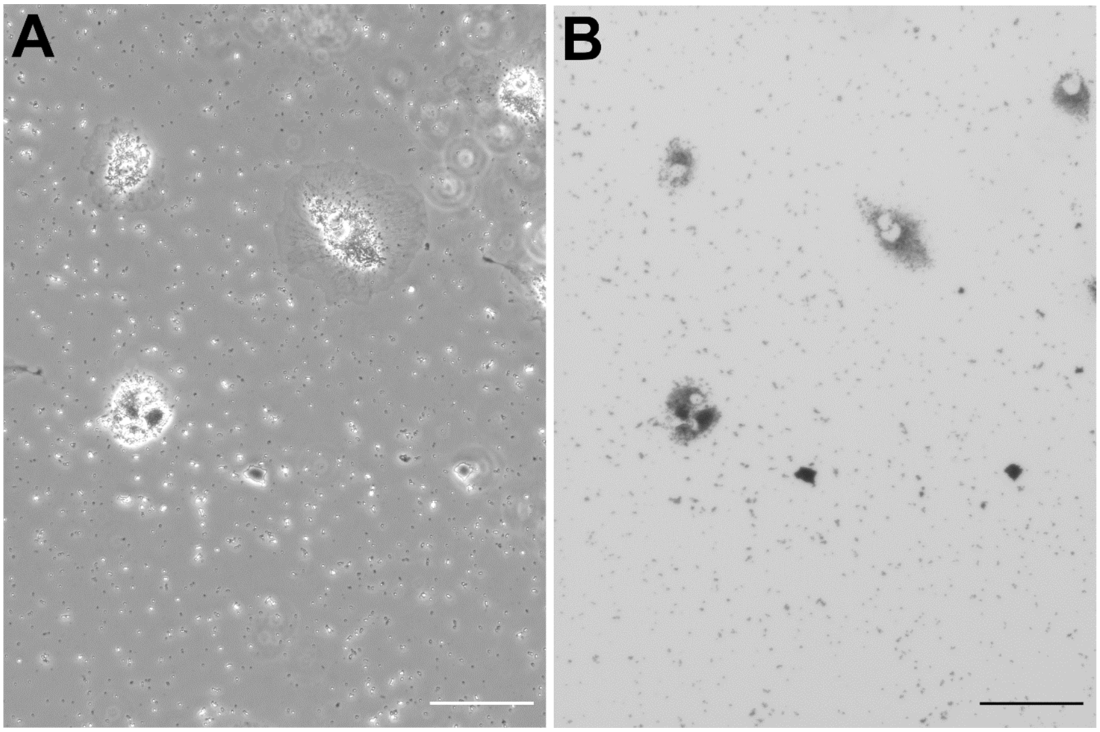

Figure S1. Phase contrast (**A**) and DIC (**B**) images of hRPE cells on day 3 in culture. The attached to the surface RPE cells spread significantly in size by forming lamellipodia (**A**), lose gradually the pigment (melanin granules or melanosomes) (recognized in B as dark dotes) and expel them into the culture solution (seen in A and B as dotes around the cells). The pigmentation of spread RPE cells is still present/conserved) and accumulated in the central part of cell body (**B**). Scale bars are 50 µm.

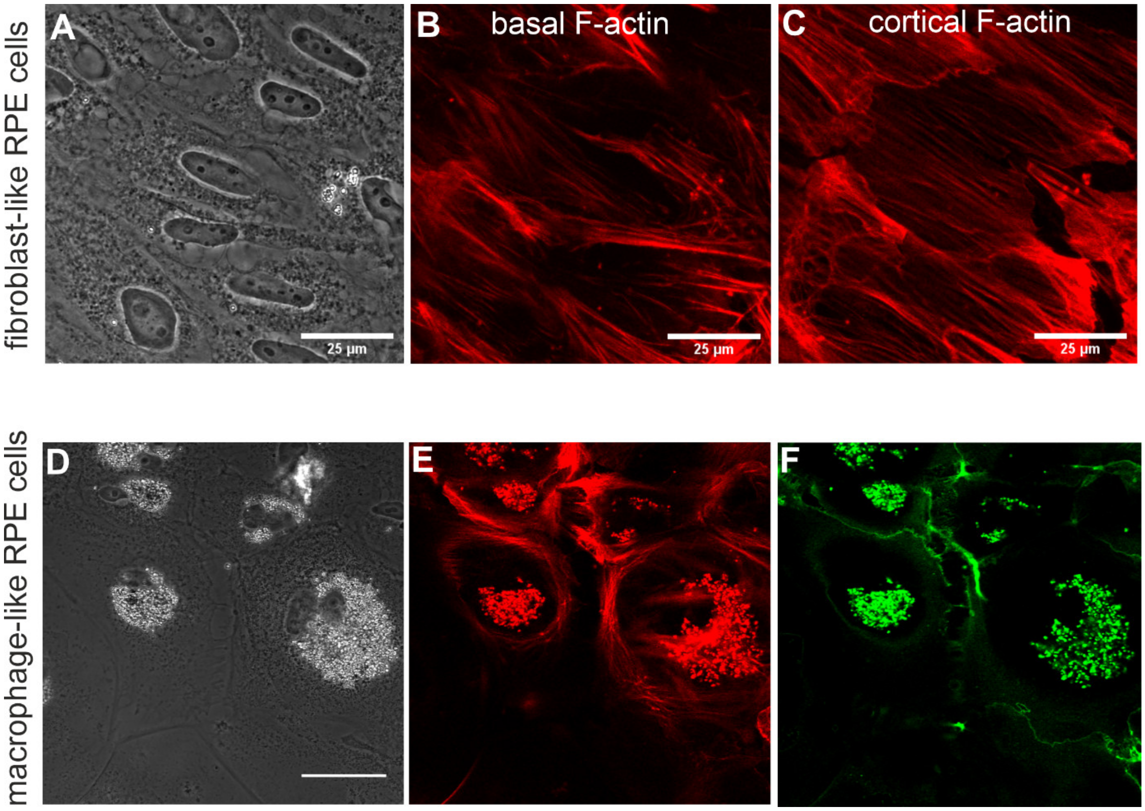

Figure S2. Simultaneously acquired phase contrast (**A**, **D**) and immunofluorescence images of fibroblast-like (upper panel) and pigmented macrophage-like RPE cells (lower panel). (**B**) and (**C**) show basal and cortical F-actin networks obtained from projection of corresponding z-stack images. (**E**) and (**F**) demonstrate melanosome autofluorescence in red-orange (excitation with 540-nm light) and green (excitation with 488-nm light) of macrophage-like RPE cells, which were dual labelled with rhodamine phalloidin (F-actin) and concavalin A (Con A) conjugated with fluorescein isothiocyanate (FITC) dye. Scale bar in (**D**) is 50 µm.

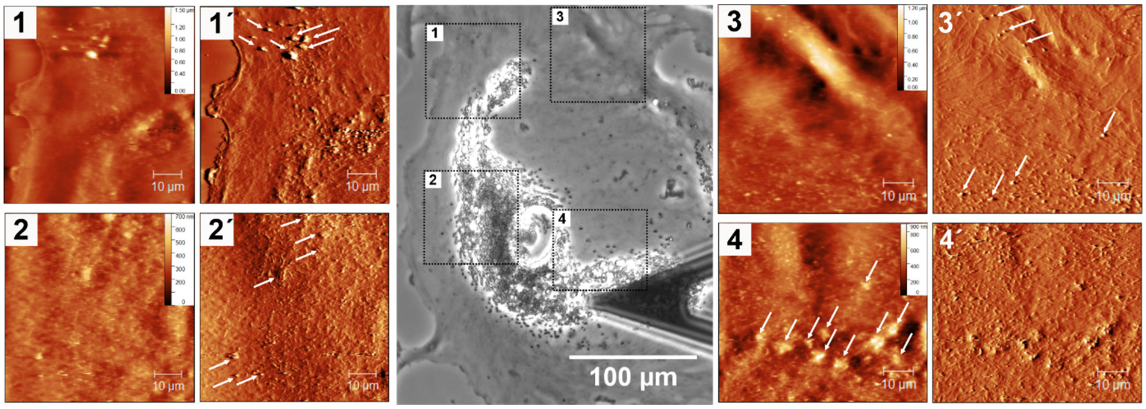

Figure S3. Phase contrast image (in the middle) of one huge macrophage-like cell with AFM (height and deflection) images collected on four different places on cell surface (**1**–**4**). Filamentous structure corresponding to the cortical cytoskeleton with stable protrusions can be clearly identified in deflection images (**1**´–**4**´)**.** Bound (entering) into the cell membrane (arrows in 1´ and 3´) and phagocyted melanosomes (arrows in 2´ and 4) can be recognized as well.
